# Ethical, Legal, and Social Implications of Symptom Checker Apps in Primary Health Care (CHECK.APP): Protocol for an Interdisciplinary Mixed Methods Study

**DOI:** 10.2196/34026

**Published:** 2022-05-16

**Authors:** Anna-Jasmin Wetzel, Roland Koch, Christine Preiser, Regina Müller, Malte Klemmt, Robert Ranisch, Hans-Jörg Ehni, Urban Wiesing, Monika A Rieger, Tanja Henking, Stefanie Joos

**Affiliations:** 1 Institute of General Practice and Interprofessional Care University Hospital Tübingen Tübingen Germany; 2 Institute of Occupational and Social Medicine and Health Services Research University Hospital Tübingen Tübingen Germany; 3 Institute of Ethics and History of Medicine University Tübingen Tübingen Germany; 4 Institute of Applied Social Science University of Applied Science Würzburg-Schweinfurt Würzburg Germany; 5 Faculty of Health Science Brandenburg University of Potsdam Potsdam Germany

**Keywords:** symptom checker apps, self-diagnosis, self-triage, digitalization in primary care, general practitioners, symptom checker, app, mobile app, primary care

## Abstract

**Background:**

Symptom checker apps (SCAs) are accessible tools that provide early symptom assessment for users. The ethical, legal, and social implications of SCAs and their impact on the patient-physician relationship, the health care providers, and the health care system have sparsely been examined. This study protocol describes an approach to investigate the possible impacts and implications of SCAs on different levels of health care provision. It considers the perspectives of the users, nonusers, general practitioners (GPs), and health care experts.

**Objective:**

We aim to assess a comprehensive overview of the use of SCAs and address problematic issues, if any. The primary outcomes of this study are empirically informed multi-perspective recommendations for different stakeholders on the ethical, legal, and social implications of SCAs.

**Methods:**

Quantitative and qualitative methods will be used in several overlapping and interconnected study phases. In study phase 1, a comprehensive literature review will be conducted to assess the ethical, legal, social, and systemic impacts of SCAs. Study phase 2 comprises a survey that will be analyzed with a logistic regression. It aims to assess the user degree of SCAs in Germany as well as the predictors for SCA usage. Study phase 3 will investigate self-observational diaries and user interviews, which will be analyzed as integrated cases to assess user perspectives, usage pattern, and arising problems. Study phase 4 will comprise GP interviews to assess their experiences, perspectives, self-image, and concepts and will be analyzed with the basic procedure by Kruse. Moreover, interviews with health care experts will be conducted in study phase 3 and will be analyzed by using the reflexive thematical analysis approach of Braun and Clark.

**Results:**

Study phase 1 will be completed in November 2021. We expect the results of study phase 2 in December 2021 and February 2022. In study phase 3, interviews are currently being conducted. The final study endpoint will be in February 2023.

**Conclusions:**

The possible ethical, legal, social, and systemic impacts of a widespread use of SCAs that affect stakeholders and stakeholder groups on different levels of health care will be identified. The proposed methodological approach provides a multifaceted and diverse empirical basis for a broad discussion on these implications.

**Trial Registration:**

German Clinical Trials Register (DRKS) DRKS00022465; https://tinyurl.com/yx53er67

**International Registered Report Identifier (IRRID):**

DERR1-10.2196/34026

## Introduction

### Background

The number of health-related software in consumer and research-oriented apps is increasing rapidly. Symptom Checker Apps (SCAs) are one example for health-related software that could have a major impact on health systems on all levels. SCAs process medical symptoms that users enter by applying algorithms and databases with medical information [1]. Based on these symptoms, SCAs generate a list of probable causes and suggest medical follow-up actions (eg, wait at home, see a doctor, go to the emergency room). The Google Play Store already lists 249 apps (retrieved on March 24, 2021) for the key words “symptom checker” [2]. Some SCA manufacturers advertise that they have implemented artificial intelligence [1,3,4] and big data as the basis of their apps. For example, as described by Richens et al [5], the SCA “Babylon” uses a causal machine learning approach based on a Bayesian approach combined with counterfactual inference. The presented algorithm achieved expert clinical accuracy for a test set of clinical vignettes [1]. It, however, remains unclear how well it performs in real-life situations. Although there have already been strong claims from ethical research that emphasize the significance of criteria such as transparency, trustworthiness, agency, and responsibility for artificial intelligence–driven decision support systems such as SCAs [6-8], not all manufacturers using artificial intelligence consider these criteria, and it is mainly untransparent how user data are processed and algorithms are trained.

The regulation of SCAs varies in different countries. In some countries, SCAs are effectively unregulated (eg, Australia [9]); in others, SCAs are regulated but with a period of “enforcement discretion” without active regulatory (eg, US Food and Drug Administration [10]). In the European Union, there is a transition between regulation through manufacturer “Declaration” of conformity to legislation toward a model of regulator audit of compliance with standards, including the formal reporting and evaluation of specified forms of clinical data and surveillance trends [11].

Symptom checkers are low-threshold tools that can be accessed with a suitable electronic device with internet access such as a smartphone and are available as apps or as browser versions. Users must be able to interact with technical devices and to interpret the SCA’s output to utilize them properly. This could lead to a disadvantage of specific population groups, for example, older adults, people with disabilities [12], or people with limited economic resources [13]. Some SCAs exclude specific user groups for symptom analysis, for example, pregnant women, children, older adults, and patients with specific comorbidities [14].

A recent study by Aboueid et al [15] investigated the intention to use symptom checkers for self-triage and revealed 5 profiles by using a latent class analysis: tech acceptors, tech rejectors, skeptics, unsure acceptors, and tech seekers. Tech seekers, which were described as participants who have positive perspectives related to SCA functionality and artificial intelligence but do not perceive to have access to the technology, showed the highest odds to use SCAs. However, the sample investigated only students aged between 18 and 34 years [15].

Although some users found that SCAs are useful tools for self-diagnosis and even reported positive health effects [16], other users had problems providing and interpreting concrete information on symptom time patterns or severity [17].

SCAs recommend actions and probable causes for the entered symptoms through their output if the output is incongruent with the users’ experience or if expectation discrepancies arise [18] and may initiate unnecessary health care–seeking behavior [19].

In terms of their medical value and validity, commercially available SCAs still have problems with accurate triage (determining a user’s medical condition based on their input and recommending the optimal health-related actions for the user). Several studies showed that SCAs often suggested risk-averse action recommendations [20-22]. SCA diagnostic and triage accuracy is still limited and was even less reliable in nonurgent scenarios, which are common in primary care [20,23]. A recently published study compared the performance of SCAs (n=8) with the performance of telephone consultation with the general practitioner (GP) (n=7) by using case vignettes (n=100). GPs outperformed SCAs on all assessed outcomes (accuracy, condition suggestion, appropriateness, and safety of urgency advice). The comparison was limited to telephone consultations and did not comprise direct patient-physician contact. Another recent study compared the performance of SCAs to that of medical laypersons using clinical vignettes and found that most laypersons outperformed the majority of SCAs, even though SCAs detected emergency cases more reliably than the laypersons [23].

In high-performing health care systems, inaccurate triage can cause preventable costs and increase the risk of unnecessary procedures that could lead to avoidable risks for patient’s safety [3,24]. However, in structurally weak regions with restricted access to medical care, SCAs can provide a first-line assessment that otherwise would not be available [25].

In summary, the potential risks of the use of SCAs (exclusion of users, stress, and induction of health-seeking behavior) contrast the advertised opportunities of SCAs such as patient empowerment and better health care for underserved regions. There is a substantial gap in the literature concerning the effect of SCAs on different health care systems, different levels of health care (microlevel, mesolevel, and macrolevel [26]) within these systems, and the system’s different participants (users, nonusers, and health care providers). If SCAs become more widely used, their ethical, legal, social, and systemic impacts on these levels and participants must be better understood despite complex interactions and methodological challenges. In this study, we aim to clarify the ethical, legal, social, and systemic impacts of SCAs on users, nonusers, GPs, the primary health care systems, and their work by means of an independent, empirical, integrated multi-perspective, and multidisciplinary discussion.

### Objectives

Owing to the lack of systematic research of SCAs in primary health care, the recent study uses an explorative hypothesis-generating approach in which the abovementioned discussion is informed by 4 foci of interest and the study aims, as stated in the following section.

#### Focus 1: Ethical, Legal, and Social Issues of SCA Use

We aim to identify the ethical, social, and legal subjects in the recent scientific literature on SCAs (eg, usage linked to inequities in health care, patient autonomy, modification of role concepts and agency).

#### Focus 2: SCA Epidemiology, Users, Nonusers, and Predictors of Use

Our results will contribute to describing the user group and nonuser group of SCAs in more detail. The degree of use of SCAs in Germany will be derived, and predictors for SCA use will be identified. Moreover, vulnerable groups that might be disadvantaged through the implementation of SCAs will be described.

#### Focus 3: Patterns and Impact of SCA Use and the User Level

As a goal for the users’ perspective, a comprehensive description of SCA use will be derived. This will comprise the assessment of requirements that are fulfilled or unfulfilled by SCA use. Additionally, we aim to identify the possible risks associated with SCA use and assess how users handle SCA information and action recommendations. SCA effects on user agency, health and eHealth literacy, well-being, and self-care will also be observed.

#### Focus 4: Impact of SCA Use on Health Care Systems and Health Care Workers

We will specially focus on considering the impact of SCAs from a health system perspective by assessing the following: changes in the patient-physician relationship, strategies of handling preinformed patients, changes in the role concept of physicians and requirements of GP, as well as potential psychosocial risks and demands and perceived work stress resulting from these changes.

## Methods

### Study Design

This study’s areas of focus and corresponding study phases will be conducted by multidisciplinary partners from the areas of social medicine, ethics and medical history, legal studies, general practice, sociology, occupational health medicine, and health services research. We will investigate SCAs that offer self-triage and action recommendations for medical laypersons. The project partners will co-develop study materials, subsequently discuss results, and conduct method workshops throughout the 3 years. During the final study year, a series of workshops will include participants of the preceding study parts. This workshop series during the final year is led by a social scientist with comprehensive experience of working in cross-disciplinary research and holding method workshops. The workshops will also serve as internal quality control and monitoring. An advisory board is continually informed about the progress and the results of the study phases. Members of the advisory board will be recruited from different contexts and disciplines. The advisory board will meet annually to give feedback on the research process, preliminary results, and the dissemination of the latter.

### Ethical Considerations

**Figure 1:**
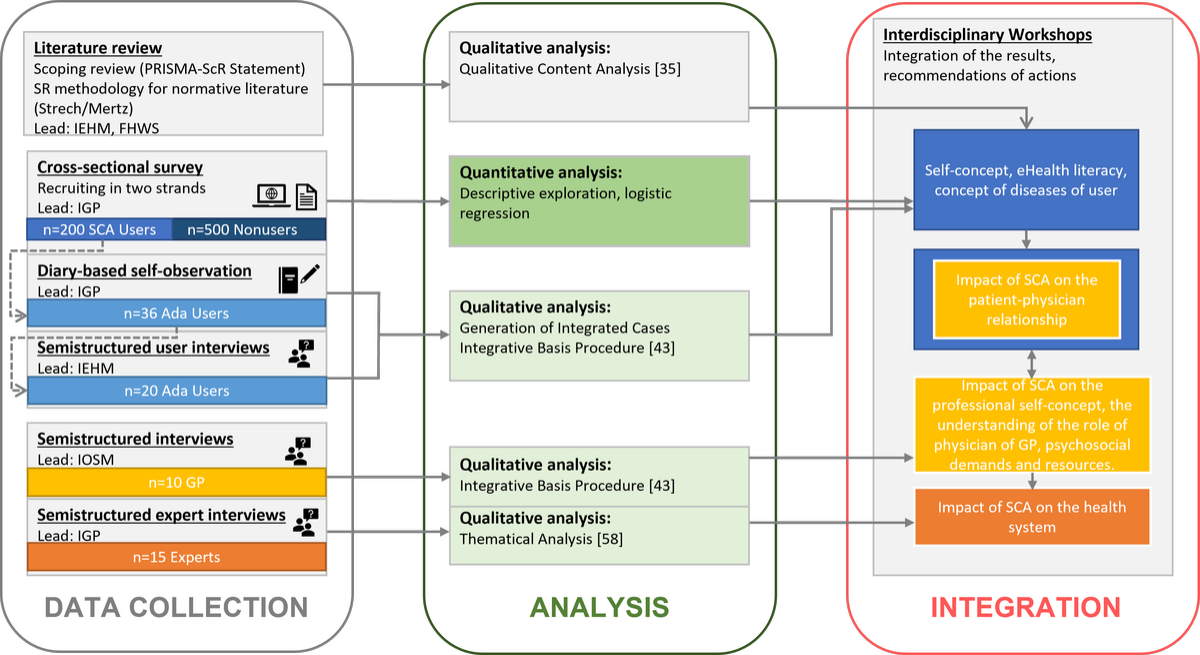
Overview of the study design, research partners and analysis methods. IEHM: Institute of Ethics and History of Medicine, University Tübingen, IGP: Institute of General Practice and Interprofessional Care, University Hospital Tübingen, IOSM: Institute of Occupational and Social Medicine and Health Services Research, University Hospital Tübingen, FHWS: University of Applied Sciences Würzburg-Schweinfurt, SCA: Symptom Checker App.

The German Federal Ministry of Education and Research funds the project for 3 years (Grant 01GP1907A). Ethical approval for this study was obtained from the ethics committee of the University of Tübingen (ID: 464/2020BO). This study will be conducted in accordance with the Declaration of Helsinki. Study participants will be informed thoroughly about the study and their rights, and written informed consent will be obtained from all study participants. Other research ethics requirements such as data protection will be diligently considered. The general study design and the involved research partners are outlined in Figure 1.

### Study Course

Four main data sources will be considered, each representing a specific stakeholder group of SCAs: representative sample of the German population (divided into SCA user and nonuser), GP, and health care experts. We define SCA users as participants that have used SCAs at least once, and nonusers are participants who never used SCAs. Data collection and analysis methods will comprise qualitative and quantitative approaches.

The different methods are applied to the data sources in 4 interconnected study phases, each representing one of the four main foci of interest of the study: a literature review (study phase 1, lead: Institute of Ethics and History of Medicine, University Tübingen [IEHM], Institute of Applied Social Science, University of Applied Science Würzburg-Schweinfurt), a representative survey of SCA user and SCA nonuser (study phase 2, lead: Institute of General Practice and Interprofessional Care, University Hospital Tübingen [IGP]), an SCA user diary-based self-observation combined with individual semistructured interviews (study phase 3, lead: IEHM), and lastly, single semistructured interviews with GPs and health care experts (study phase 4, lead: Institute of Occupational and Social Medicine and Health Services Research, University Hospital Tübingen [IOSM] for GP interviews, lead: IGP for health care experts interviews). For this study protocol, we will follow the Good Reporting of A Mixed Methods Study (GRAMMS) [27] checklist. Preferred Reporting Items for Systematic reviews and Meta-Analyses extension for Scoping Reviews (PRISMA-ScR) [28,29], STROBE (STrengthening the Reporting of OBservational studies in Epidemiology) [30], SRQR (Standards for Reporting Qualitative Research) [31], and GRAMMS [28] checklists will be applied during the project process. The PRISMA-ScR, STROBE, and SRQR guidelines will be applied on specific study phases. The GRAMMS [28] guideline will be used in the context of mixed methods approaches and in the integration of results. In the following sections, the 4 study phases and their connections are described in detail.

### Study Phase 1: Literature Review

The first study phase will comprise a comprehensive literature review that will assess the existing research on SCAs and their impact on primary care. The aim of this study phase is to gain a clearer picture of the state of science of SCAs, considering the ethical, legal, social, and systemic (eg, risks, potentials) impacts of SCAs. A literature search, oriented on scoping review (ScR) methodology, will be conducted and reported according to the PRISMA-ScR statement [28,29]. In recent years, the ScR methodology has been adopted and further developed for the field of bioethics, which is characterized by normative research questions. When analyzing argumentative literature, adjustments need to be made to the “classic” ScR methodology [32-34]. Key terms will be defined for the search strategy regarding the research questions, and databases covering the relevant dimensions (biomedical, ethical, social, and legal) are selected (Web of Science, PubMed [Medline], Belit/Ethmed, ProQuest, SowiPort, GESIS, Philpapers, Juris, BeckOnline, etc). The inclusion criteria comprise either the mention of an ethical, legal, or social issue or a combination of this terms related to SCAs as a digital or mobile app, targeting medical laypersons that support the assessment of symptoms and self-triage or one of both aspects. Apps for health care professionals or other experts, as well as SCAs, which focus on a single health condition or certain groups of diseases were excluded. To present the full spectrum of ethical, legal, social, and systemic impacts literature relevant to the review question, not only argument-based but also empirical literature was included. Publications on SCAs that were written in English or German and published until 2020 were included in the review. Journal articles, contributions to anthologies, reports, case series, letters to editors, opinions, commentaries, and conference papers were also included; web-based blogs have not been considered. Three researchers screened the identified literature via hand and database search and discarded publications not meeting the inclusion criteria. Publications were analyzed by 2 authors using the method of qualitative content analysis proposed by Kuckartz [35].

### Study Phase 2: Cross-sectional Survey of SCA Users and SCA Nonusers

#### Structure

A survey will be used in a case-control design. The questionnaire was piloted with 5 participants and will take approximately 15-25 minutes for participants to complete. The survey will comprise different evaluated scales (for further details, see Multimedia Appendix 1) and sociodemographic variables as well as specific questions to the usage of SCAs. This study phase will be conducted and reported according to the STROBE statement [30]. Owing to the limited amount of literature on SCA users and nonusers, pilot interviews with 2 SCA users and 1 SCA expert will be conducted to ensure a meaningful concept selection for the survey. Simultaneously, concepts will be derived from existing literature that is connected to the use of health apps and could reveal the potential characteristics of the user group such as eHealth Literacy [36], personality [37,38], hypochondria [39], self-efficacy [40,41], and need for cognition [42]. Affinity for technology [43], satisfaction with the GP [44], and overall life satisfaction [45] will also be considered. Moreover, we will assess the perceived usefulness of SCAs and requirements for SCAs in open-ended questions of the survey since this might be a central aspect of acceptance of SCAs as the Technology Acceptance Model (TAM) induces. The TAM was already introduced by Davis [46] in 1989 and is based on the theory of reasonable action and the theory of planned behavior. The TAM (and its further expansions) is still one of the most prevailing models to examine factors affecting a users’ acceptance of new technologies [47]. The model assumes a mediating function of perceived ease of use and usefulness in association with system characteristics and system usage [47].

#### Sample and Recruitment

The sampling process was conducted from November 2020 until the end of June 2021. A case-control design using 2 recruitment strands is planned. An a priori power analysis using PASS 2020 (v20.0.3, NCSS) revealed a sample size of 375 (β=.8, α=.05, nuisance parameter=0.2; n_user_=188, n_nonuser_=188) for an odds ratio of 2.5. The targeted odds ratio corresponds to a small-to-medium effect of Cohen *d* [48] and was selected because we consider that this will be an effect size that contributes a meaningful explanation of variance in the logistic regression. As this study has an explorative character, we could not derive theory-driven assumptions for a multivariate logistic regression. Hence, we based the power analysis on a univariate logistic regression. We will use a univariate logistic regression to identify meaningful predictors for the usage of SCAs and will moreover set up a multivariate model that includes all the identified predictors. Our multivariate model will be the first proposal and will need further research to confirm the univariate predictors in a multivariate model.

The sample will be composed of different recruiting strands to achieve a representative sample. In the first strand, German citizens will be contacted via mail to participate in the survey. The intended recipients will be representatively selected by an external partner (T + R Dialog Marketing and Acxiom). Further participants will be recruited via mailing lists of the University of Tübingen and the University Hospital of Tübingen, social media, and cooperating GP practices. After 3 months, the representativity of this sample will be checked. If the return rate is too low or if certain groups are not sufficiently represented, there will be additional recruiting via the proposed channels.

The second strand of the sampling process aims to integrate symptom checker users only. We expect only a small number of symptom checker users. To ensure a sample size of n_user_=188, a targeted recruitment via social media advertisements and the social media channels of the University Hospital of Tübingen will be conducted.

Inclusion criteria in general are the ability to give consent and German language skills of at least B1 of the Common European Framework of References for Languages. Participants of the second recruitment strand can only be included if they have experience with SCAs.

#### Analysis

The level of use for SCAs, awareness of SCAs, and general interest in SCAs will be described using the first recruitment strand with descriptive statistics.

Following the case-control design, SCA users from the first and second recruiting strand will be matched with nonusers (matched controls) from the first recruitment strand. Significant predictors will be extracted with a logistic regression. A correction for multiple testing will be applied. The recent versions of SPSS (IBM) and R statistic (R Core Team) will be used for the analyses.

### Study Phase 3: Diary-Based Self-observation Combined With Semistructured User Interviews

#### Structure

Study phase 3 investigates SCA users and their usage patterns and effects of SCAs on individuals. A specific SCA (Ada app) was chosen by the study team as an example since it is considered one of the most prevalent SCAs in Germany. Following web-based training, participants will engage in a diary-based self-observation. During the observation time of 6 weeks, participants will document their daily usage and nonusage of the Ada app. Next, individual semistructured user interviews are performed with the diary study participants. The interviews allow participants to reflect on values, concepts, and knowledge gaps. This allows a supplemental exploration of the experiences recorded in the diaries. The user interviews in this study part will be conducted and reported according to SRQR statement [31].

#### Sample and Recruitment

For this study phase, 50 Ada users will be recruited from the SCA user strand of phase 2 and, if needed, additionally via social media. Considering a dropout rate of 30%, a sample size of 36 is assumed. Of these participants, 15 will be recruited for single semistructured interviews using maximum variation sampling. Sampling will consider the content of the diary-based self-observation, usage behavior of the app, medical indication, and socioeconomic factors. Sample size calculations of the interview phases are based on the 5D model of *information power* by Malterud et al [49].

All participants will receive web-based training on the self-observation period. The diary will be used to document symptoms and events, as well as other expected influencing factors such as stress or quality of life. Furthermore, it will offer structured questions about the use of SCAs and enable the participants to write down their own reports or short “field notes.” Thus, participants will record and describe their experience, and how they dealt with action recommendations, appearing problems, emotions, etc. These notes will be used as a basis for the following semistructured interviews. The interviews will be conducted via video call, audio-recorded, and transcribed verbatim by a researcher from the IGP and the IEHM. The users will receive financial compensation both for the interviews and the participation in the member check meeting described below.

#### Analysis

The diary-based self-observation and the interview transcripts will be analyzed and integrated into cases. Triangulating the self-observation diary data with interview transcripts provides both prospective (longitudinal) and retrospective (narrative) insights. Quantitative results of the diaries (frequency of use, use of health care, symptoms, etc) will be considered as prospective observational outcomes and will be analyzed quantitatively. The user diaries give the opportunity to record detailed situational experiences—feelings and thoughts that probably cannot be remembered or recreated during an interview without them. However, the interviews will provide in-depth reports on values, concepts, gaps in knowledge, etc, that tend to remain invisible in the diaries. The qualitative analysis via Kruse’s integrative basic procedure [43] will provide an overview of the recurring themes and patterns within each case as well as between cases. At the same time, it will allow a more holistic consideration of the data such as the analysis of semantics, grammatical structures, and metaphors to reveal latent meanings and the way users “make sense” of the app and derive meaning and understanding of the recorded events [50]. The qualitative analysis of the study is supported by MaxQDA [51]. A member check with participants of the interviews is planned, in which results of the cases are presented to study participants to enhance rigor.

The aim of the quantitative analysis is to identify meaningful predictors for the use of SCAs, taking the longitudinal data structure into account. A hierarchical model with 2 levels will be performed. Level two will comprise the daily measurements and will be nested in level one, which comprises the participants. The quantitative analysis of the diaries will be performed using a recent version of Microsoft Excel [52] and R Statistics (R Core Team).

### Study Phase 4: Semistructured Interviews With GPs and Health Care Experts

#### Structure

The fourth study phase investigates the possible effects of SCAs on health care delivery, health care providers (module A), and the health care system (module B). As primary care is most affected by patients’ usage of SCAs, we will interview GPs in module A. We will gain more insights into patients’ usage of SCAs and similar application results in potential psychosocial demands, resources, and perceived work-related stress [53], especially regarding workload, work content, work organization, and social environment [54]. Module B aims to deliver a multi-perspective view on possible effects of SCAs on the health care system. To fulfill this aim, we will conduct interviews with health care experts with different backgrounds to assess the state of science of SCAs in practice from a multi-perspective standpoint. Moreover, we aim to identify which potential experts see for the future use of SCAs and to derive quality criteria for SCAs. This study phase will be conducted and reported according to SRQR statement [31].

#### Sample and Recruitment

In module A, the sample will consist of 10 GPs in Germany. We aim to build a heterogeneous sample regarding the GP (age, gender, and race) and their practices (structure and location of the practice, main patient clientele, and availability of web-based services). Sample size calculations of the interview phases in modules A and B are based on the 5D model of *information power* by Malterud et al [49]. An interview guide [55] will be developed, containing questions about preinformed patients and diagnosis in general, questions about SCAs, and similar apps with the example of the Ada app in particular. It will be developed by the IOSM and the help of feedback from the other project partners. The IOSM will apply various forms of sampling such as snowball sampling in the established networks of the IOSM and web-based research to ensure the stated heterogeneity of the sample. Two researchers of the IOSM will conduct the interviews mostly via video call due to the ongoing pandemic. The interviews are expected to last about 45 minutes and will be audio-recorded.

In module B, 10-15 experts on health care systems will be interviewed. Experts will be recruited consecutively and comprise politicians, information technology developers for medical software, patient advocates, representatives from jurisdiction, medical associations, and health insurances. We assume that 10-15 interviews will provide sufficient information power [49]. Each interview guide will be tailored to the respective expert. Possible topics are the implementation of SCAs, recent issues with SCA requirements, and how SCAs influence different players in health care. The interview guides will be developed by the team of the IGP with input from the other project partners. The experts will be contacted via already existing research networks of the project partners. One researcher of the IGP will conduct the interviews mostly via video call. The interviews are planned to last about 45-90 minutes and will be audio recorded.

The expert interviews will provide an information background on the *status quo* of SCAs in health care as well as ideas for future developments. This background is important for the discussion of user experiences (Study phase 3) and for the patient-physician relationship (integrated study phases 3 and 4). All participants will receive financial compensation both for the interviews and the participation in the member check meeting.

#### Analysis

All interviews will be transcribed. In module A, the interviews will be analyzed with reflexive thematical analysis by Braun and Clark [56]. As already stated, this method is used to understand how interview participants “make sense” of their experiences. This allows insight into tacit changes in self-concepts, implicit values psychosocial demands and resources, and perceived work-related stress. Additionally, it allows to conduct an analysis of themes and content provided by the interview partners. The IOSM team will individually evaluate each interview and compare the interviews to analyze common patterns. To ensure quality control and richness of analysis, each interview will be analyzed by 2 researchers, and preliminary results will be continuously discussed with additional researchers from the joint project. The interviews in module B will be analyzed with reflexive thematical analysis by Braun and Clark [56], as we aim to collect and structure the overarching themes and their various dimensions. The same measures for quality control, as described in module A, will be applied. For additional quality control, a member check with interview participants of phase 4 is planned: preliminary results will be presented, and participants will be invited to give critical feedback, which will be integrated into the further analysis.

## Results

The project started in March 2020.

### Study Phase 1: Literature Review

The literature review on ethical, legal, social, and systemic impacts of SCAs was completed in December 2021 and will be published in 2022.

### Study Phase 2: Cross-sectional Survey of SCA Users and SCA Nonusers

The data collection for study phase 2 was finished in July 2021 (n=1074); the publication of the results is planned for 2022.

### Study Phase 3: Diary-Based Self-observation Combined With Semistructured User Interviews

For study phase 3, data collection of the user diaries (n=48) was completed in October 2021, user interviews were conducted in February and March 2022, and publication of the results is planned for 2022.

### Study Phase 4: Semistructured Interviews With GPs and Health Care Experts

The recruitment of experts and GPs (study phase 4) is still ongoing and will be finished in 2022.

## Discussion

### Principal Findings

This protocol describes an interdisciplinary, mixed methods, multiphase research program to comprise the impact of SCAs on the 3 levels (microlevel, mesolevel, and macrolevel) of the health care system [26]. The main findings of the recent exploratory study will be an overview of the ethical, legal, social, and systemic impacts literature on SCAs, epidemiology data of SCAs (as degree of use in Germany, predictors for SCA usage considering user characteristics), requirements of SCA use from a user perspective, a description of SCA user behavior, and a comprehensive assessment of the perspective of GPs and health care experts on SCAs.

Digital health care innovations and their impact on the health care system is a prevailing topic in times of digitalization and the increasing demand of health care professionals such as GPs. In contrast to apps for specialists, user-accessible apps such as SCAs are an unknown variable in the development of health care delivery in the future. Conflicting claims of medical, ethical, and social advantages or disadvantages of SCAs characterize the current state of the debate. Apparently paradoxical effects, as undermining trust in the patient-physician relationship on the one hand, improved exchange on the other hand, may be coexisting or representing different perspectives in scenarios that require further description. The conflicting information about ethical, legal, social, and systemic impacts requires more empiric data to inform and deepen the debate [57].

Little is known about the psychosocial demands and resources of GPs in this context. SCAs are attributed to result in an overuse as well as in an underuse of health care resources. Finally, existing health inequalities may be improved or worsened by their impact. Based on an ScR of the ethical, social, and legal literature, the project will provide evidence, which of these contradicting assumptions is confirmed by the empirical study of the user experiences in the case study at hand. Using SCA as an example of consumer-oriented digital innovations, this study aims to research and integrate questions that are important for the general debate on digital transformation: what is known about the topic? How widespread is the phenomenon? How do users apply the innovation? How does that modulate their behavior and impact their health care usage? How do negotiations with health care providers play out? Which regulatory legislation is necessary? What are the implications for the physicians in particular and the health care system as a whole? However, only collecting data from 1 stakeholder’s perspective without considering possible interactions will generate blind spots. Thus, the main challenge is to consider different stakeholders’ perspectives, wants and needs, and to engage in a transparent debate on the current dynamic developments.

### Strengths and Limitations

The projects’ concept integrates different data sources and methods from the very start. The multidisciplinary, multiphase design, and the methods and skills mix of the study partners create a scenario in which methodological strengths are complimentary and perspectives can be negotiated. For example, by limiting recruitment to a single SCA (the Ada app) in the qualitative study phases, we are able to focus the analysis [49]. At the same time, through the representative survey, these qualitative results can be put in a broader context, which will contribute to implications for the health care system. Another strength is the immanent consideration of the user perspective by combining a survey, user diary, and user interviews. In integrating relevant perspectives and plotting study phases to converge in integrated workshops, we present an approach for integrated research in ethics, social, and health sciences. This is possible owing to the long-standing cooperation between all involved partners and previous positive experiences in common projects.

This research project also has limitations: we will not investigate how SCAs perform in terms of medical accuracy. Further, we will not be able to observe direct interaction of patients and physicians in the context of SCAs and see how SCAs influence the patient-physician relationship directly. Moreover, we assessed user and nonuser characteristics through subjective user rating rather than objective measurements (eg, overall health rating of participant). Lastly, we will not directly investigate the effect of SCAs on health care utilization. We will, however, assess parameters considering utilization reported by participants.

### Conclusions

This study offers an opportunity for multidisciplinary research: it considers different research perspectives and methodologies from ethics, legal, social, health care, and medical science and integrates them in 1 study process. We are confident that this will lead to new insights for the use of SCAs and digitalization in health care while providing a novel methodological approach for integrated research in health care digitalization.

## Appendix

Multimedia Appendix 1 Evaluated scales that were used in the survey of study phase 2.

## Abbreviations

GP: general practitioner
GRAMMS: Good Reporting of A Mixed Methods Study
IEHM: Institute of Ethics and History of Medicine, University Tübingen
IGP: Institute of General Practice and Interprofessional Care, University Hospital Tübingen
IOSM: Institute of Occupational and Social Medicine and Health Services Research, University Hospital Tübingen
PRISMA-ScR: Preferred Reporting Items for Systematic reviews and Meta-Analyses extension for Scoping Reviews
SCA: Symptom Checker App
ScR: scoping review
SRQR: Standards for Reporting Qualitative Research
STROBE: STrengthening the Reporting of OBservational studies in Epidemiology
TAM: Technology Acceptance Model
