# Supplementary figures and images for: Ethical, Legal, and Social Implications of Symptom Checker Apps in Primary Health Care (CHECK.APP): Protocol for an Interdisciplinary Mixed Methods Study
Source: JMIR Res Protoc. 2022 May 16;11(5):e34026. doi: 10.2196/34026 (PMC9152714; doi:10.2196/34026)

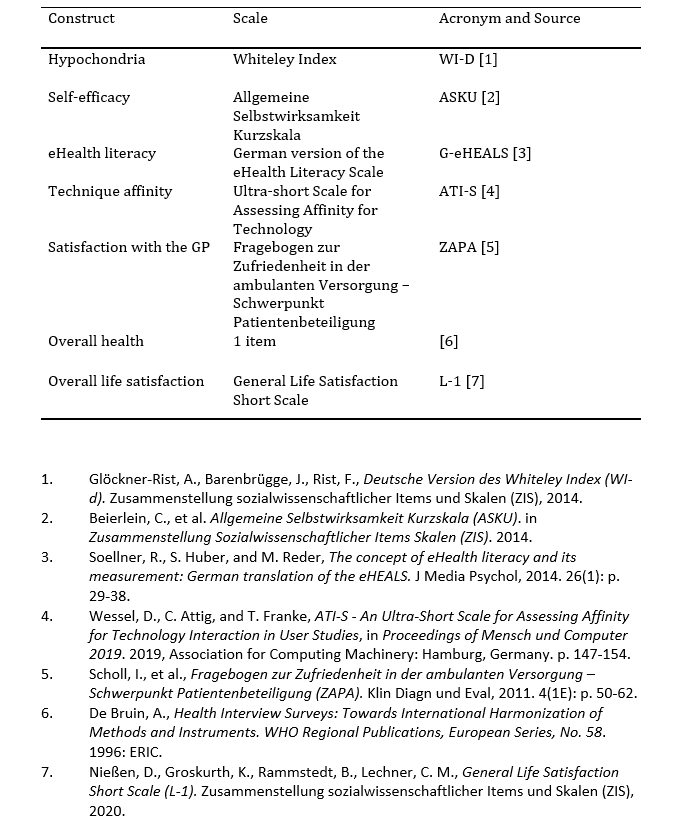

Supplement: Multimedia Appendix 1 [file resprot_v11i5e34026_app1.png]
